# P-TEFb Regulates Transcriptional Activation in Non-coding RNA Genes

**DOI:** 10.3389/fgene.2019.00342

**Published:** 2019-04-24

**Authors:** Heeyoun Bunch, Hyeseung Choe, Jongbum Kim, Doo Sin Jo, Soyeon Jeon, Sanghwa Lee, Dong-Hyung Cho, Keunsoo Kang

**Affiliations:** ^1^Department of Applied Biosciences, College of Agriculture and Life Sciences, Kyungpook National University, Daegu, South Korea; ^2^Department of Transcriptome & Epigenome, Macrogen Incorporated, Seoul, South Korea; ^3^Institute of Life Science and Biotechnology, College of Natural Science, Kyungpook National University, Daegu, South Korea; ^4^Department of Life Science, College of Natural Science, Kyungpook National University, Daegu, South Korea; ^5^Department of Microbiology, College of Natural Sciences, Dankook University, Cheonan, South Korea

**Keywords:** non-coding RNA, RNA polymerase II promoter-proximal pausing, P-TEFb, gene expression regulation, transcriptional elongation

## Abstract

Many non-coding RNAs (ncRNAs) serve as regulatory molecules in various physiological pathways, including gene expression in mammalian cells. Distinct from protein-coding RNA expression, ncRNA expression is regulated solely by transcription and RNA processing/stability. It is thus important to understand transcriptional regulation in ncRNA genes but is yet to be known completely. Previously, we identified that a subset of mammalian ncRNA genes is transcriptionally regulated by RNA polymerase II (Pol II) promoter-proximal pausing and in a tissue-specific manner. In this study, human ncRNA genes that are expressed in the early G_1_ phase, termed immediate early ncRNA genes, were monitored to assess the function of positive transcription elongation factor b (P-TEFb), a master Pol II pausing regulator for protein-coding genes, in ncRNA transcription. Our findings indicate that the expression of many ncRNA genes is induced in the G_0_–G_1_ transition and regulated by P-TEFb. Interestingly, a biphasic characteristic of P-TEFb-dependent transcription of serum responsive ncRNA genes was observed: Pol II carboxyl-terminal domain phosphorylated at serine 2 (S2) was largely increased in the transcription start site (TSS, -300 to +300) whereas overall, it was decreased in the gene body (GB, > +350) upon chemical inhibition of P-TEFb. In addition, the three representative, immediate early ncRNAs, whose expression is dependent on P-TEFb, metastasis-associated lung adenocarcinoma transcript 1 (*MALAT1*), nuclear enriched abundant transcript 1 (*NEAT1*), and X-inactive specific transcript (*XIST*), were further analyzed for determining P-TEFb association. Taken together, our data suggest that transcriptional activation of many human ncRNAs utilizes the pausing and releasing of Pol II, and that the regulatory mechanism of transcriptional elongation in these genes requires the function of P-TEFb. Furthermore, we propose that ncRNA and mRNA transcription are regulated by similar mechanisms while P-TEFb inhibition unexpectedly increases S2 Pol II phosphorylation in the TSSs in many ncRNA genes.

**One Sentence Summary:** P-TEFb regulates Pol II phosphorylation for transcriptional activation in many stimulus-inducible ncRNA genes.

## Introduction

Gene expression regulation is the most fundamental and crucial event to ensure development, growth, and homeostasis in living organisms. Genes on the DNA double strand are expressed to RNAs and some of them to protein molecules through transcription and translation. The human genome, composed of approximately 3 billion nucleotides, encodes approximately 20,000 annotated protein-coding genes^1^ (International Human Genome Sequencing Consortium, 2004). It is estimated that about 70% of the human genome is transcribed and yet only < 2% of the transcripts are mRNAs that are translated into proteins (Fatica and Bozzoni, 2014; Palazzo and Gregory, 2014). This indicates the vastness of non-protein coding RNA genes in mammalian cells. In addition to their large number, more and more non-coding RNAs (ncRNAs) have been found to exert important, diverse cellular functions. Therefore, understanding the functions and mechanisms of ncRNAs has become essential in biology and medicine.

Despite the fact that a vast majority of ncRNAs have no known function, the critical roles of different ncRNAs have been continuously discovered since XIST was found to mediate the X-chromosome inactivation process in 1992 (Brockdorff et al., 1992; Brown et al., 1992; Gilbert et al., 2000). In particular, ncRNAs regulate transcription of protein-coding genes (Espinoza et al., 2004; Carrieri et al., 2012; Yoon et al., 2012; Bunch, 2018). Gene regulation by ncRNAs is mediated through direct recruitment/interaction with transcriptional activators (or repressors) and epigenetic modification at the transcription level (Popov and Gil, 2010; Cao, 2014; Sarma et al., 2014; Zhang et al., 2014; Tsoi et al., 2015; Singh et al., 2018). For example, maternally expressed 3 (MEG3) facilitates the recruitment of p53 on its target genes (Zhou et al., 2007), and both MALAT1 and NEAT1 are abundant in actively transcribed genes (West et al., 2014), implying transcriptional activation assisted by these two factors. XIST interaction with Polycomb proteins is known as a key event for causing the epigenetic insulation of an X chromosome, thus silencing the gene expression (Brockdorff, 2013; Lee and Bartolomei, 2013). For the post-transcription level, some ncRNAs including an 18-mer originating from the *TRM10* locus, β-site APP cleaving enzyme-1- antisense transcript (BACE1-AS), TINCR, and a variety of microRNAs (miRNAs) regulate protein synthesis and target mRNA turnover by modulating the productivity of ribosomes or by stabilizing or destabilizing mRNA (Faghihi et al., 2010; Yoon et al., 2012; Kretz et al., 2013).

ncRNAs are not intermediate molecules, like mRNAs, that are translated into proteins (Cech and Steitz, 2014). ncRNAs perform a variety of cellular functions, regulating molecular interactions between macromolecules (nucleic acids and proteins) in the cell. The expression of ncRNAs is dependent on transcription, RNA processing/maturation, and RNA turnover. The regulation of ncRNA transcription is thought to resemble the protein coding gene transcription. This assumption is attributed to the similarities between ncRNA and mRNA synthesis. Many ncRNAs are transcribed by Pol II and are capped at the 5′ end and polyadenylated at the 3′ end (Beaulieu et al., 2012) and are spliced (Tilgner et al., 2012; Soreq et al., 2014) and post-transcriptionally modified (Fu et al., 2014). A number of long non-coding RNAs (lncRNAs) with sizes greater than 200 bp, are divergently transcribed from protein coding genes, and some of these ncRNA-protein coding gene couples are coordinately or interdependently transcribed (Core et al., 2008; Sigova et al., 2013; Wu and Sharp, 2013). In addition, we have shown that a majority of lncRNAs (>1000 bp) harbor Pol II paused in the promoter-proximal site (Bunch et al., 2016; Bunch, 2018). Pol II pausing is the way to achieve synchronized and instantaneous gene expression upon gene activation. From what has been learned from the transcriptional mechanisms of protein-coding genes, prevalent Pol II pausing in ncRNA genes suggests a critical checkpoint between the early and processive elongation of Pol II for ncRNA transcription (Core et al., 2008; Adelman and Lis, 2012; Bunch et al., 2014; Bunch et al., 2016; Bunch, 2018). It also emphasizes the inducibility of ncRNA genes by transcriptional activators upstream and in the proximity of TSS for gene activation (Rahl et al., 2010; Zobeck et al., 2010; Bunch et al., 2016; Bunch, 2017).

Pol II pausing is stabilized or released by pausing regulators, pausing, or pause-release factors, respectively (Brown et al., 1996; Wu et al., 2003; Lee et al., 2008; Jonkers and Lis, 2015; Chen et al., 2018; Fitz et al., 2018). In the case of protein-coding genes, those with Pol II pausing are expressed little in the ground state where Pol II is stably associated with the nascent RNA and the DNA template in the promoter proximal region, +25–+100 from the TSSs in metazoans (Adelman and Lis, 2012; Liu et al., 2015; Bunch, 2016). The pausing is mediated and stabilized by different factors and elements including DRB sensitivity inducing factor (DSIF), negative elongation factor (NELF), tripartite motif-containing 28 (TRIM28), Pol II-associated factor 1 (PAF1), GAGA factor, +1 nucleosome, and nucleic acid (DNA or RNA) secondary structure (Wu et al., 2003; Lee et al., 2008; Gilchrist et al., 2010, 2012; Bunch et al., 2014; Jonkers and Lis, 2015; Zhang and Landick, 2016; Chen et al., 2018). In addition, recent studies have suggested that Pol II pausing is the short-duration stage for an individual Pol II (Krebs et al., 2017; Steurer et al., 2018). Pol II pausing in a gene is kept steady before productive elongation because of the rapid turnover of consecutive Pol II molecules in the pausing site. Although Pol II pausing apparently halts transcription during the inactive state of gene expression, it conditions and prepares the nascent RNA, transcription machinery, and nucleosome architecture for processive elongation, immediately following the reception of transcription-activating signal in the promoter region (Adelman and Lis, 2012; Bunch et al., 2015; Jonkers and Lis, 2015; Bunch, 2017). Therefore, Pol II pausing is a prerequisite step for productive transcription in a number of stimulus-inducible genes.

In protein-coding genes, P-TEFb is an important protein factor for Pol II pausing regulation and active transcription (Lis et al., 2000; Jonkers and Lis, 2015; Yu M. et al., 2015; Aj et al., 2016; Lu et al., 2016; Ebmeier et al., 2017). In *HSP70*—a model gene to study Pol II pausing regulation—the master transcriptional activator, heat shock factor 1 (HSF1), is activated by phosphorylation, binds to *heat shock element* (HSE) in the promoter, and then recruits P-TEFb to the TSS (Lis et al., 2000; Bunch et al., 2014). On the other hand, some report that an inactive complex of P-TEFb including HEXIM1 and 7SK snRNA regulates Pol II pausing, and the release of CDK9 and Cyclin T1, an active P-TEFb complex from the inactive complex promotes pause release (D’Orso, 2016). In a number of stress/stimulus-inducible protein-coding genes, P-TEFb phosphorylates DSIF, NELF, and Pol II CTD at serine 2 (S2 Pol II) upon transcriptional activation, and this phosphorylation is required for Pol II to be released from the pausing site to resume transcription (Ping and Rana, 2001; Peterlin and Price, 2006; Adelman and Lis, 2012; Lu et al., 2016). During transcriptional activation, phospho-S2 Pol II becomes accumulated in the gene body, which is a bona-fide indicator of processive Pol II elongation (Hintermair et al., 2012; Bunch et al., 2014, 2015).

The P-TEFb has been characterized to a lesser extent in ncRNA transcription. Besides the similarities and sharing elements between protein-coding and ncRNA gene transcription, our previous study has shown that many ncRNA genes are stimulus-inducible, harboring Pol II pausing (Bunch et al., 2016; Bunch, 2018). In this study, therefore, we hypothesized that P-TEFb plays an important regulatory role in ncRNA transcription and aimed to evaluate the function of P-TEFb and S2 Pol II phosphorylation in ncRNA transcription. Importantly, our data showed that many serum-inducible ncRNA genes show P-TEFb-dependent transcriptional activation. In the presence of flavopiridol (hereafter, flavo), an inhibitor of P-TEFb, however, a number of ncRNA genes increased phospho-S2 Pol II occupancies in the TSS despite decreased occupancies in the gene body. This biphasic effect of P-TEFb inhibition has not been reported for protein-coding genes and is thus apparently unique for ncRNA genes, and may involve additional kinase(s) regulating S2 Pol II phosphorylation in ncRNA transcription. In addition, by probing phospho-S2 Pol II, a subset of serum-inducible or serum-repressed ncRNA genes was identified and characterized. Among the serum-inducible ncRNA genes, *MALAT1, NEAT1*, and *XIST* are immediate early ncRNAs expressed in the early G_1_ phase. The transcription and serum-inducibility of these clinically important ncRNAs requires the kinase activity of P-TEFb and utilizes TATA binding protein (TBP).

## Materials and Methods

### Cell Culture and Experimental Conditions

HEK293 cells (obtained from ATCC) in the study were grown in DMEM (Corning, NY, United States) supplemented with 10% FBS (Gibco, United States) and 1% Penicillin/Streptomycin (P/S, Thermo Fisher, United States) solution. For serum induction experiments, HEK293 cells were grown to about 80% confluence. The cells were incubated in DMEM including 0.1% FBS and 1% P/S solution for 17.5 h and then induced using serum by incubating in DMEM supplemented with 18% FBS and 1% P/S solution. After serum induction, cells were collected at corresponding time points listed in figures. For inhibition experiments, HEK293 cells were incubated in the 0.1% serum media for 17.5 h. The media was exchanged with the 0.1% serum media with Flavopiridol (Sigma Aldrich, Cat. F3055, United States) at a final concentration of 1 μM in 0.1% DMSO (Sigma Aldrich, United States). The cells were incubated with the inhibitor for 1 h before serum induction with 18% serum media including the inhibitor for 15 min in the same concentration as in the pre-incubation. Control cells were prepared side-by-side using DMSO only.

### Reverse Transcription Quantitative Polymerase Chain Reaction

RNA molecules longer than 18 nt were extracted using a miRNeasy mini kit (Qiagen, Germany) as instructed by the manufacturer. For quantitative PCR, 600 or 700 ng of RNA was converted to cDNA by reverse transcription using a Promega Reverse Transcription System (Promega, Cat. A3500, United States) or Toyobo ReverTra Ace^®^ qPCR RT Master Mix (Toyobo, Japan), according to the manufacturers’ instructions. PCR was performed using the equal amount of resultant cDNAs and indicated primers through GoTag DNA polymerase (Toyobo, Japan) or Platinum Tag DNA Polymerase High Fidelity (Invitrogen, United States) under thermal cycling under the following conditions: 2 min at 94 or 95°C followed by 25 cycles of 20 or 30 s at 94 or 95°C, 30 s at 55°C, and 1 min at 68 or 72°C. Primer sequences are provided in the Supplementary Table 1.

### Chromatin Immunoprecipitation and PCR

The ChIP-PCR experiment was conducted following the Abcam X-ChIP protocol with mild modifications (Bunch et al., 2014, 2015). Cell lysis buffer included 5 mM PIPES (pH 8.0), 85 mM KCl, 0.5% NP-40, and fresh protease inhibitors described above. Nuclei lysis buffer including 50 mM Tris-Cl (pH 8.0), 10 mM EDTA, and 1% SDS was added before sonication. Sonication was performed at 25% amplitude for 30 s with 2 min intervals on ice (Vibra-Cell Model VCX 130, Sonics & Materials, Inc.) and was optimized to produce DNA segments ranging between +100 and +1,000 bp on a DNA gel. Antibodies used in IP were S2 phosphorylated Pol II antibody (5 μg/each IP) from Abcam ab5095 and TBP antibody (3 μg/each IP) and CDK9 antibody (5 μg/each IP) from Santa Cruz Biotechnology sc-421 and sc-13130, respectively. After IP and reverse-cross-linking, DNA was purified through a Qiagen PCR purification kit. Input DNAs were quantified for quantitative PCR analysis. PCR was performed as described above using Platinum Tag DNA Polymerase High Fidelity (Invitrogen, United States): pre-denaturation for 2 min at 94°C, 30 cycles of denaturation at 94°C, annealing at 55°C for 30 s, and extension at 68°C for 30 s. The antibodies listed above have been validated for the relevant species and applications, and the validation is provided on the manufacturers’ websites.

### Immunoblotting and Gel Electrophoresis

HEK293 cells grown in 6-well plates for Western blots and SDS polyacrylamide gel electrophoresis (SDS-PAGE) were washed with cold PBS twice and scraped in RIPA buffer (Cell Signaling, Cat. 9806, United States). Protein concentration in each sample was measured through Bradford assay using Bio-Rad Protein Assay Dye Reagent Concentrate (Bio-Rad #5000006) and spectrophotometry at 595 nm (Tecan Sunrise^TM^ Absorbance Microplate Reader, Switzerland). From the measured protein concentration, a total of 15 μg of proteins per sample was loaded on 7% SDS-PA gels, blotted onto nitrocellulous membrane, and probed for Pol II, phospho-S2 Pol II, and α-Tubulin using corresponding antibody (Pol II, Cell Signaling 2629S; phospho-S2 Pol II, Abcam ab5095; α-Tubulin, Santa Cruz ac-8035) in Western blot assay. For SDS-PAGE, a total of 20 μg of proteins per sample was loaded onto 10% SDA-PA gels and then stained using Coomassie Brilliant Blue Reagent (Bio-Rad, United States).

### Fluorescence-Activated Cell Sorting

Flow cytometric analysis was performed to determine the presence of cell cycle status. The cells were harvested by trypsinisation and fixed with ice-cold 70% ethanol for 1 h at 4°C. For fluorescence-activated cell sorting (FACS) analysis, the cells were washed with PBS twice and suspended in 1 ml of cold DAPI solution, then incubated on ice for 30 min for analysis by a flow cytometer (MACSQuant^®^ Analyzer, Miltenyi Biotec). At least 50,000 cells were addressed and the data were analyzed by using FCS Express (De Novo Software).

### ChIP-seq

#### Library Preparation and Sequencing

Illumina libraries were prepared using a Beckan-Coulter SPRIworks system and sequenced on an Illumina HiSeq2000 using a 40 nt single-end read. *Sequencing data QC*- Single-end reads (40 bp) were verified for the sequence quality with FastQC (version 0.10.0). Before starting analysis, Trimmomatic (version 0.32) (Bolger et al., 2014) was used to remove the bases with low base quality. *Aligning read to the reference genome*- The cleaned reads were aligned with the human genome (UCSC hg19) using Bowtie (version 1.1.2) (Langmead et al., 2009), allowing up to two nucleotide mismatches to the reference genome per seed and resulting in acquiring only uniquely mapped reads. Mapped data (SAM file format) were performed sorting and indexing using SAMtools (version 1.2.1) (Li et al., 2009). The read counts in each ncRNA around the transcription start site (TSS) were calculated with the BEDtools multicov program (version 2.20.1) (Quinlan and Hall, 2010). *Discovering candidate peak region*- Peaks were called in the aligned sequence data using the model-based analysis of ChIP-seq (MACS version 2.1.1.20160309) (Zhang et al., 2008) with a *q*-value (FDR adjusted *p*-value for multiple testing) cut-off of 0.05. The algorithm empirically models the length of ChIP-Seq fragments from the sequence data, considering local genomic biases for the analysis of distribution of mapped reads. ChIPseeker (version 1.10.3) (Yu G. et al., 2015), an R/bioconductor package for annotating enriched peaks identified from ChIP-seq data, was used to identify nearby genes and transcripts from the peaks obtained from MACS.

#### Differential Profile Analysis

**FIGURE 1:**
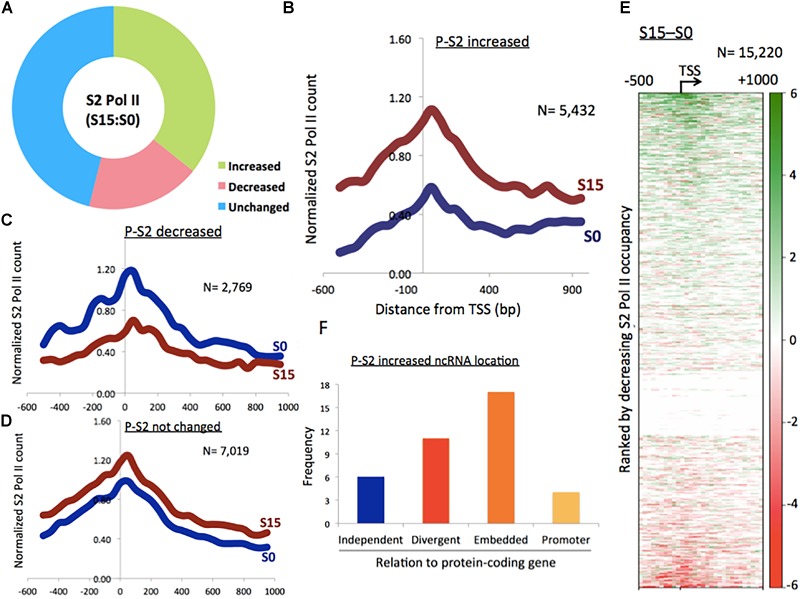
Phosphorylation of Pol II CTD at serine 2 in ncRNA genes. **(A)** Proportion of phospho-S2 Pol II-increased (35.7%), -decreased (18.2%), and unchanged (46.1%) ncRNA genes upon serum induction in HEK293 cells. **(B)** Phospho-S2 Pol II profile showing a subset of ncRNA genes with increased phospho-S2 Pol II over twofold upon serum induction in S0 and S15 (*n* = 5432). P-S2, phospho-S2 Pol II. **(C)** Phospho-S2 Pol II profile showing the ncRNA genes with decreased S2 Pol II over twofold upon serum induction (*n* = 2769). **(D)** Phospho-S2 Pol II profile showing the ncRNA genes with less than twofold change upon serum induction (*n* = 7019). **(E)** Heat map of phospho-S2 Pol II in ncRNA genes (*n* = 15,220). S15–S0. **(F)** Genome localization of ncRNA genes with dramatically increased phospho-S2 Pol II (>150-fold change) upon serum induction, relative to neighboring protein-coding genes.

**FIGURE 2:**
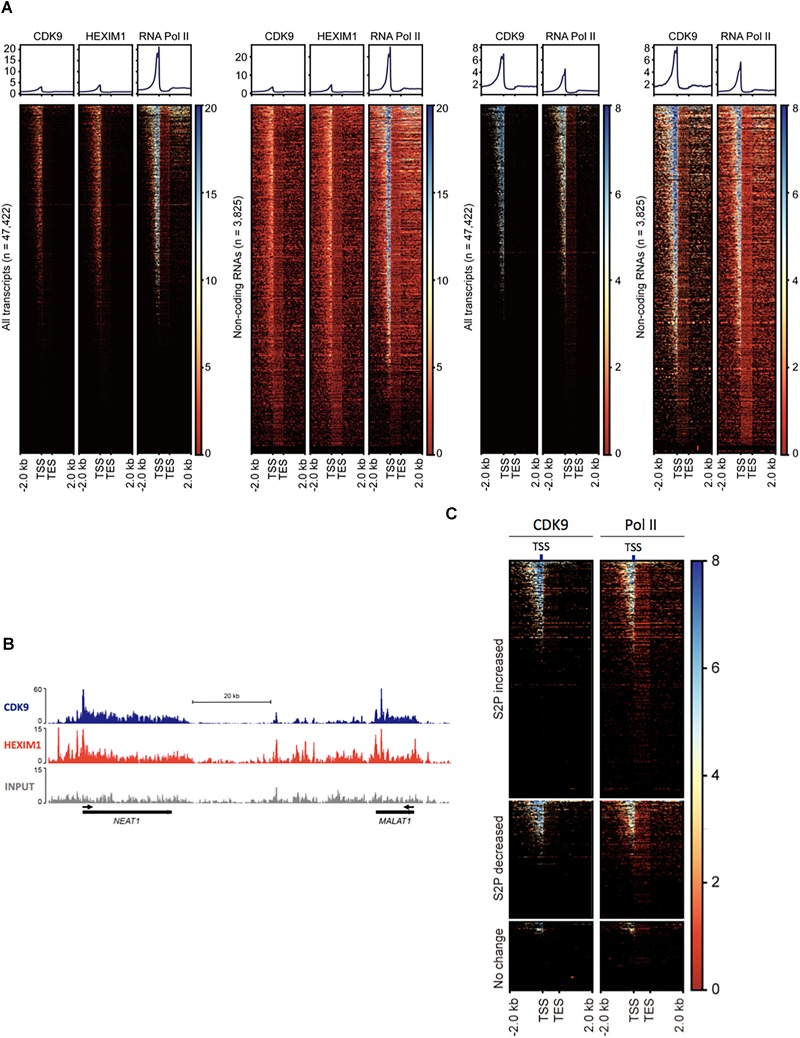
P-TEFb association with ncRNA genes. **(A)** Heatmaps of normalized Pol II, CDK9, and HEXIM1 occupancies in all genes (*n* = 47,422) and ncRNA genes (*n* = 3825) in melanoma (A375, left two sets) in HEK293T cells (right two sets). TES, transcription end site. **(B)** Chromosome views of CDK9 and HEXIM1 at *NEAT1* and *MALAT1*, two representative genes. The TSSs are shown in black arrows. **(C)** Heatmaps of normalized Pol II and CDK9 in phospho-S2 Pol II (S2P)-increased, -decreased, and unchanged ncRNA genes upon serum induction; *n* = 5432 (fold change > 2), 2769 (fold change > –2), and 2110 (–1 < fold change < 1), respectively. S2P-increased or -decreased ncRNA genes display co-localization of Pol II and CDK9 in the TSS, whereas these factors are deprived in S2P-unchanged genes.

The read count value was normalized by the TMM method in edgeR (Robinson et al., 2010). Statistical significance of the signal data was determined by fold change and exactTest in which pair-wise tests were conducted for differential signal between S2 Pol II, S0 and S15 for the negative binomially distributed counts. *Visualization of binding profiles*- Heatmaps (Figure 1E, 3A) were generated using deepTools2 (computeMatrix and plotHeatmap function) (version 2.5.4) (Ramirez et al., 2016). In order to confirm the coverage, each ncRNA gene, in the chromosome view (Figure 4B), was drawn using an R/bioconductor package Gviz. In Figure 2, CDK9, HEXIM1, and Pol II ChIP-seq data in NCBI Gene Expression Omnibus under accession numbers GSE68052 (A375 cells) (Tan et al., 2016) and GSE51633 (HEK293T cells) (Liu et al., 2013) were downloaded and processed using the Octopus toolkit (Kim et al., 2018). Heatmaps were generated using deepTools (Ramirez et al., 2014) with default parameters. Integrative genomics viewer (IGV) (Robinson et al., 2011) was used to capture genomic views of CDK9 and HEXIM1 binding profiles on the ncRNA genes of interest.

## Results

### Many ncRNA Genes Accumulate Phospho-S2 Pol II During the Cell Cycle Transition to Early G_1_ Phase

We queried whether Pol II CTD phosphorylation at S2 occurs in transcriptionally activated ncRNA genes and, if so, to what extent. A number of protein-coding genes are transcriptionally activated during the G_1_ phase (Selvaraj and Prywes, 2004). Some protein-coding genes that are critically expressed in the early G_1_ phase are called immediate early genes (Lau and Nathans, 1985). These genes essentially function in memory formation, cell growth and proliferation, and are often implicated in cancers (Bahrami and Drablos, 2016). *In vivo*, these genes can be synchronized in the G_0_ phase through serum starvation and then can be released to enter the G_1_ phase through serum replenishment (Bunch et al., 2015).

We utilized this method to activate ncRNA genes that are expressed in the early G_1_ phase. After serum starvation (S0) followed by 15 min of serum replenishment (S15), human embryonic kidney 293 (HEK293) cells were harvested, and phospho-S2 Pol II was monitored using chromatin immune-precipitation-sequencing (ChIP-seq). Peaks were called in the aligned sequence data using the model-based analysis of ChIP-seq (MACS) with a *q*-value (FDR adjusted *p*-value for multiple testing) cut-off 0.05. It was observed that approximately 35.7% (*n* = 5432) of a total of 15,220 ncRNA genes increased phospho-S2 Pol II more than twofold upon serum induction (Figure 1A,B and Supplementary Data 1). Phospho-S2 Pol II decreased over twofold and unchanged ncRNA genes were 18.2% (*n* = 2769) and 46.1% (*n* = 7019) of the total ncRNAs included in our analysis, respectively (Figure 1C,D and Supplementary Data 1). A heatmap with the 15,220 ncRNA genes generated by subtracting S0 from S15 shows the patterns of phospho-S2 Pol II occupancy changes in the TSSs and gene bodies (Figure 1E). These data showed that S2 Pol II phosphorylation is up- or down-regulated in a large number of ncRNA genes (53.9%), and a majority of these ncRNA genes are enriched with phospho-S2 Pol II in the early G_1_ phase.

Some ncRNA genes that significantly increased (*n* = 34) or decreased (*n* = 10) phospho-S2 Pol II over 150-fold in S15 are summarized in Table 1. Genomic locations of most of the increased genes displayed a geometrical relation with other protein-coding or ncRNA genes: 18% of the S2 Pol II increased genes are independent and 82% are divergent to (gene gap < 3000 bp), embedded within, or in the promoter (<3000 bp from TSS) of neighboring genes (Figure 1F and Table 1). On the other hand, all the phospho-S2 Pol II decreased genes are embedded in protein-coding genes (Table 2).

**Table 1:** ncRNA genes with increased S2 Pol II in the G_0_-G_1_ transition.

ncRNA	Fold increase (S0 vs. S15)	Location relative to neighboring protein-coding genes
SNORD160	221.33	KIF2C embedded
LOC1019285	207.11	Divergent to GLRA3
LINC01276	207.11	FOXP4 promoter
BRWD1-IT2	200.01	BRWD1 embedded
HAS2-AS1	200.01	HAS2 divergent
SNORD143	192.90	SEC31A embedded, THAP9 divergent
SAPCD1-AS1	192.90	VWA7 embedded
MIR6892	185.79	FAM131B divergent
LOC1066995	178.68	DYNC1LI2 divergent
MIR4426	178.68	Overlap with RPS27AP5
ASTN2-AS1	178.68	ASTN2 embedded and divergent
MIR6857	178.68	SMC1A embedded
MIR450B	178.68	Independent
MIR450A1	178.68	Independent
LINC01135	164.47	JUN divergent
LINC01762	164.47	ncRNA divergent
STARD13-AS	164.47	STARD13 embedded
LOC1019270	164.47	PLEKHA3 promoter
MIR130B	164.47	ncRNA promoter
CHRM3-AS1	157.36	CHRM3 embedded
MIR4692	157.36	Complex
LINC01483	157.36	Independent
VTRNA1-1	157.36	ncRNA embedded
BAALC-AS2	157.36	BAALC divergent
MIR450A2	157.36	Independent
SPATA13-AS1	150.26	SPATA13 embedded
LINC00449	150.26	TM9SF2 embedded, divergent
MIR6777	150.26	SREBF1 embedded
MIR33B	150.26	SREBF1 embedded
MIR4785	150.26	RBMS1 embedded
LINC01018	150.26	Independent
LOC1019279	150.26	FARS2 embedded
SNORD52	150.26	C6ORF48 embedded
MIR4656	150.26	AP5Z1 convergent

**Table 2:** ncRNA genes with decreased S2 Pol II in the G_0_-G_1_ transition.

ncRNA	Fold decrease (S0 vs. S15)	Location relative to neighboring protein-coding genes
LOC1005062	-220.58	Embedded and convergent
MIR4726	-202.28	Embedded in MLLT6
TRHDE-AS1	-183.98	Embedded in TRHDE
MKLN1-AS	-165.68	Embedded in MKLN1
MIR3713	-165.68	Embedded in SCAPER
LOC1019289	-165.68	Embedded in TTC7B
DKFZP434K028	-165.68	Embedded in MYRF
ATP1A1-AS1	-165.68	Embedded and convergent
MIR4717	-156.53	Convergent to ABCA3
SHANK2-AS1	-156.53	Embedded in SHANK2

### P-TEFb Is Enriched in a Number of ncRNA Genes

Phospho-S2 Pol II formation requires the kinase function of P-TEFb (Schuller et al., 2016). For the prevalent phospho-S2 Pol II regulation, we attempted to map P-TEFb in ncRNA genes in human cells. The kinase subunit of P-TEFb, CDK9 and the regulatory subunit, HEXIM1 were located using the ChIP-seq data available in gene expression omnibus (GEO), GSM1661786, GSM1661787, and GSM1249897. The results indicated the tight association of CDK9 and HEXIM1 with ncRNA genes. In GSM1661786 (A375 cells), a total of 16,351 genes including protein-coding and ncRNA genes were identified to have CDK9 peaks over 10 (Supplementary Data 2). These were composed of 3611 ncRNA, 12,298 protein-coding, 280 pseudo, 2 rRNA, and 160 snoRNA genes (Figure 2A). In GSM1249897 (HEK293T cells), a total of 18,628 genes including protein-coding and ncRNA genes harbored CDK9 peaks > 10 (Supplementary Data 2). These comprised 2291 ncRNA, 15,845 protein-coding, 317 pseudo, three rRNA, and 172 snRNA genes. For HEXIM1 (GSM1661787, A375 cells), a total of 20,584 genes displayed peaks > 10, including 4155 ncRNA, 15,900 protein-coding, 372 pseudo, 4 rRNA, and 153 snoRNA genes (Supplementary Data 2).

The heatmaps of CDK9 and HEXIM1 using the ChIP-seq data with both A375 and HEK293T cells showed the enrichment of these components in the TSSs of ncRNA genes (*n* = 3825; Figure 2A). As shown in the metagene analyses in Figure 2A (upper graphs), the profile of the CDK9 and HEXIM1 peaks for ncRNA genes (*n* = 3825) was comparable with the one for all genes (*n* = 47,422) in both cell lines. In addition, we observed that CDK9 occupancies were overall overlapped with Pol II in ncRNA genes, which is similar to that observed in protein-coding genes. The similar peak depth of CDK9 and HEXIM1 between all and ncRNA genes is presumably due to the comparable pausing occurrence in the two groups and the involvement of P-TEFb inactive complex with these paused genes. A large number of protein-coding genes harbor Pol II pausing in metazoan cells, reportedly 30% in *Drosophila* and up to 91% in mice, and our previous study estimated that approximately 47% of mammalian lncRNA over 1000 bp in size include paused Pol II (Rahl et al., 2010; Adelman and Lis, 2012; Bunch et al., 2016). It is thus suggested that CDK9 and HEXIM are engaged with these paused Pol II in a large number of genes including both protein-coding and ncRNA genes. Chromosome views of representative ncRNA genes, *MALAT1* and *NEAT1*, depicted the localization of CDK9 and HEXIM1 (Figure 2B). These data suggest the involvement and important function of P-TEFb in the regulation of ncRNA transcription, consistent with the phospho-S2 Pol II accumulation during stimulus-inducible gene expression as shown in Figure 1. In addition, we analyzed CDK9 and Pol II occupancies in the ncRNAs in which S2 Pol II phosphorylation became increased (fold change > 2, *n* = 5432), decreased (fold change > -2, *n* = 2769), or unchanged (-1 < fold change < 1, *n* = 2110) in response to serum. As expected, phospho-S2 Pol II increased or decreased genes harbored CDK9 more abundantly in the TSS than unchanged ncRNA genes did (Figure 2C). CDK9 occupancy appears to overlap with Pol II peaks, concentrated in the promoter-proximal site. These results reinforce the important function of P-TEFb to Pol II activity and gene expression in inducible ncRNA genes.

### Flavopiridol Interferes With Phospho-S2 Pol II Accumulation in a Biphasic Manner

**FIGURE 3:**
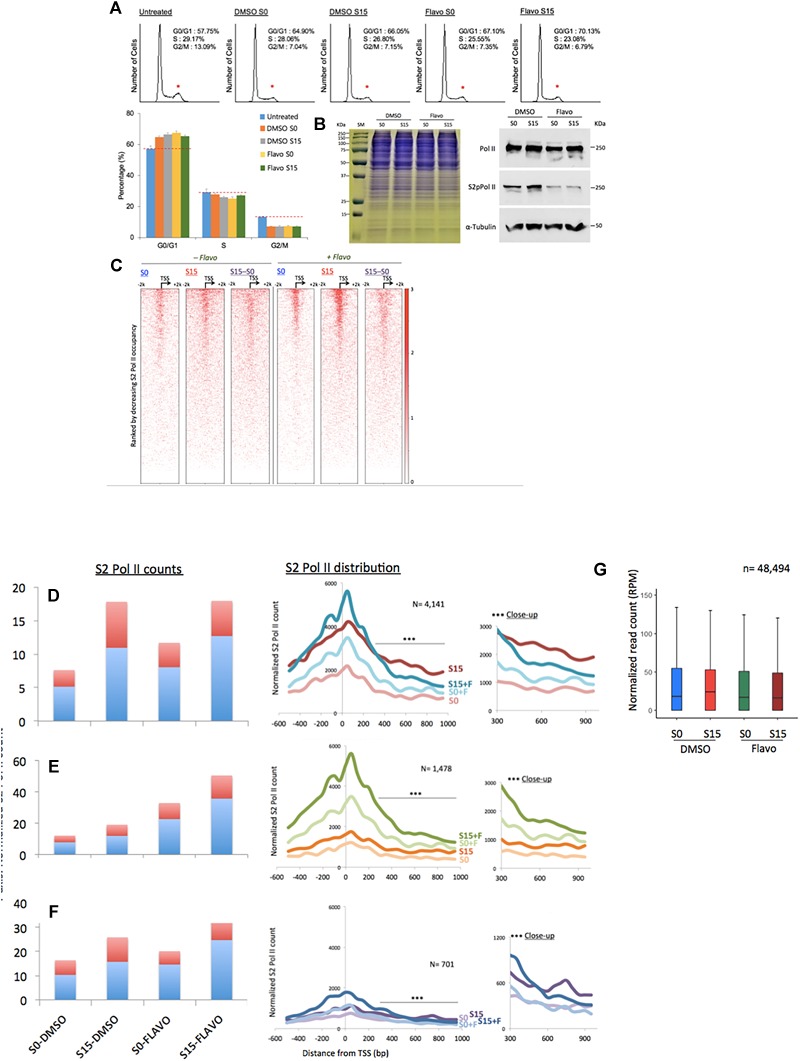
Flavopiridol effects on the transcription of ncRNA genes. **(A)** Fluorescence-activated cell sorting data showing the cell-cycle synchronization status in the samples. Untreated, the cells grown in the complete media (DMEM including 10% fatal bovine serum). Reduction in the G_2_/M phase is marked with small, red stars above the peaks. Data presented are the averages of 5 measurements. Error bars, SD (*n* = 5). **(B)** Gel electrophoresis of HEK293 cell extracts of DMSO- or flavo- (Flavo) treated cells (left). Proteins stained with Coomassie Brilliant Blue. Western blots for cell extracts probing total Pol II, S2 phosphorylated Pol II (S2pPol II), and α-Tubulin, showing decreased phospho-S2 Pol II despite the similar amounts of total Pol II in flavo-treated cells (right). The result suggested the specificity of phospho-S2 Pol II antibody and the effectiveness of flavo conditions that are used in the study. SM, size marker. **(C)** Phospho-S2 Pol II ChIP-seq. Heat maps of S2 phosphorylated Pol II in ncRNA genes (*n* = 15,520) before (S0) and 15 min after serum induction (S15) in the presence and absence of flavo in HEK293 cells. Flavo treatment results in the overall increase of phospho-S2 Pol II occupancies in the TSSs while decreasing them in the gene bodies. Serum-induced phospho-S2 Pol II increase is alleviated or enhanced in the presence of flavo (see also **B,C**). **(D)** Phospho-S2 Pol II distribution in the ncRNA genes with the decreased phospho-S2 Pol II ratio over twofold between S0 and S15 in the presence of flavo (+F) (*n* = 4141). In the bar graph, TSS (shown in light blue) is the genomic locus between –300 and +300 from the TSSs of ncRNA genes. Gene body shown in red is the downstream of +300, between +350 and +950 from the TSS. Note the increase of phospho-S2 Pol II in the TSS and gene body in S0+F in comparison with S0 (before serum induction, DMSO control). For S15 samples, phospho-S2 Pol II increase in the downstream of +350 from TSS becomes noticeably reduced in S15+F, compared to S15 (serum induced, DMSO control). In the right panel, a line with three stars indicates the area of zoom-in on right side (^∗∗∗^close-up). **(E)** Phospho-S2 Pol II profile of the ncRNA genes with the increased S2 Pol II count ratio (S15:S0) over twofold in the presence of flavo (*n* = 1478). **(F)** Phospho-S2 Pol II profile of the ncRNA genes without notable changes (0.9 < fold change < 1.1) of S2 Pol II counts upon serum induction with or without flavo (*n* = 701). **(G)** Metagene analysis of the occupancy changes of phospho-S2 Pol II (S2 Pol II) in the region between –2000 and +2000 from the TSS in protein-coding genes (*n* = 48,494).

Next, we investigated the effect of P-TEFb inhibition on ncRNA transcription. For an effective functional interference of P-TEFb, we employed a small chemical inhibitor, flavo (Chao and Price, 2001). It is noted that flavo inhibits CDK9, the kinase subunit of P-TEFb, and other CDKs including CDK1, CDK2, CDK4, and CDK6 (Chao and Price, 2001). Flavo (1 μM final concentration) was applied to the G_0_-synchronized HEK293 cells for 1 h before the cell cycle was triggered to the G_1_ phase by exchanging the media including 18% serum with flavo. Then, cell-cycle progression and gene activation were allowed for 15 min as described above. To ensure the targeted cell-cycle synchronization, the cell-cycle stages of DMSO- and flavo-treated cells were monitored through FACS. The results showed that serum starvation increases the cell population in the G_0_/G_1_ phase and reduces it in G_2_/M, compared to the control grown in the complete media without any treatment (Figure 3A). In addition, DMSO- and flavo-treatment cells similarly responded to the serum starvation and induction (Figure 3A and Supplementary Data 3).

As many ncRNA genes accumulate S2-phosphorylated Pol II upon transcriptional activation, we hypothesized that flavo, by inhibiting P-TEFb, would block the increase in phospho-S2 Pol II in ncRNA genes. We observed little difference in the protein level among the four samples of S0 and S15 in presence of DMSO or flavo, shown in the gel electrophoresis result (left, Figure 3B). Western blot assay, however, showed the global reduction of S2 phosphorylated Pol II in flavo-treated cells (Flavo S0 in Figure 3B). In addition, upon serum induction (DMSO S15), we could detect a moderate increase in phospho-S2 Pol II, whereas flavo dramatically inhibited the increase (Flavo S15). Interestingly and unexpectedly, the metagene analyses presented mixed populations of phospho-S2 Pol II profiles when the control and P-TEFb-inhibited samples were compared, with and without flavo. A heatmap shown in Figure 3C depicts the impact of the inhibition of S2 Pol II phosphorylation in all ncRNA genes (*n* = 15,220). Approximately 37% of ncRNA genes decreased or increased phosphorylated S2 Pol II over twofold in the presence of flavo (*n* = 4141 and 1478, respectively; Figure 3D,E and Supplementary Data 4). Interestingly, even for ncRNA genes with overall decreased phospho-S2 Pol II of over twofold in the presence of flavo (*n* = 4141), the drug caused a noticeable increase in S2 Pol II phosphorylation in the TSS for both G_0_-synchronized (S0) and early G_1_ (S15) cells despite the decreased serum-induced S2 Pol II accumulation in the gene body (Figure 3D). Figure 3D shows the increased S2 Pol II near the promoter-proximal site, TSS, defined as the genomic region between -300 and +300 from the TSS, in the presence of flavo. When we collected ncRNA genes with increased phospho-S2 Pol II (over two-fold) in the presence of flavo (*n* = 1478), flavo was found to dramatically increase phospho-S2 Pol II in the TSS, again for both G_0_-synchronized and early G_1_ cells (Figure 3E). In addition, the profile of phospho-S2 Pol II in this group of ncRNA genes appeared to have increased in the gene body, defined as the genomic region between +350 and +950 from TSS, with flavo (Figure 3E). For ncRNA genes with negligible changes in phospho-S2 Pol II occupancies (0.9 < fold change < 1.1, *n* = 701), a similar tendency to the first group (Figure 3D) was observed, where the overall phospho-S2 Pol II increased in the TSS and decreased in the gene body in the presence of flavo (Figure 3F).

In protein-coding genes, P-TEFb inhibition by flavo interferes with S2 Pol II phosphorylation upon serum-induced transcriptional activation. It is noted that phospho-S2 Pol II has not been mapped genome-wide with and without functional P-TEFb (e.g., +/- flavo) so far. Despite this, studies have shown an overall reduction of total and phospho-S2 Pol II, in particular, in the gene bodies of targeted genes (Rahl et al., 2010; Bunch et al., 2015). On the other hand, P-TEFb inhibition is attributable to a mild increase of total Pol II accumulation in the TSSs, probably as a result of hindered pause release/proceeding to the elongation (Jonkers et al., 2014; Steurer et al., 2018). The metagene analysis with protein-coding genes (*n* = 48,494) using our S2 phosphorylated Pol II ChIP-seq data showed a mild decrease of S2-phosphorylated Pol II in S0 and S15 cells under flavo treatment (Figure 3G). It is noteworthy that flavo increases S2 Pol II phosphorylation in the TSSs of many ncRNA genes (Figure 3C–F), a phenomenon apparently different from what has been widely considered, although not empirically verified, in protein-coding genes (Figure 3G). In contrast, S2 Pol II phosphorylation in the gene body appears to be dependent on P-TEFb function following transcriptional activation in many ncRNA genes, similar to what has been observed in protein-coding genes. This biphasic characteristic of P-TEFb inhibition effect could be unique for ncRNA genes and may indicate unknown layers of Pol II phosphorylation regulation such as unidentified kinase(s) or regulator(s) for S2 Pol II in the promoter-proximal sites of ncRNA genes.

### *MALAT1, NEAT1*, and *XIST* Gene Activation in Early G_1_ Phase

**FIGURE 4:**
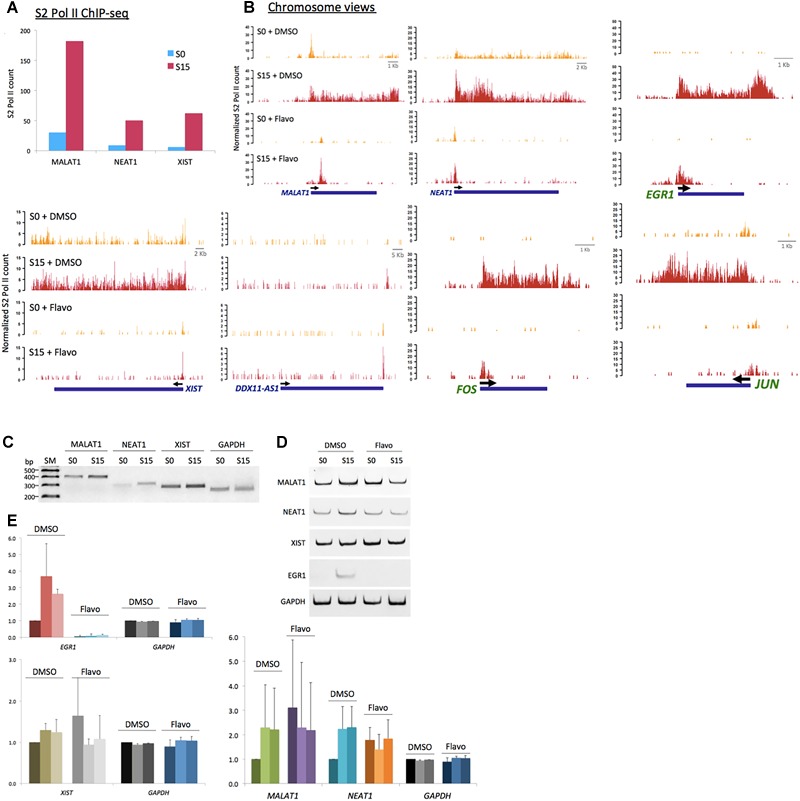
P-TEFb function in ncRNA genes, MALAT1, NEAT1, and XIST. **(A)** Phospho-S2 Pol II ChIP-seq results. Phospho-S2 Pol II counts at *MALAT1, NEAT1*, and *XIST* are increased in serum-induced HEK293 cells. **(B)** Chromosome views of phospho-S2 Pol II in *MALAT1, NEAT1*, and *DDX11-AS1* in S0 and S15 with and without flavo. *DDX11-AS1* was included as a control whose expression was not induced by serum and thus not affected by flavo in the given condition. *EGR1, JUN*, and *FOS* were included as positive controls, protein-coding genes established for P-TEFb-dependent gene activation, for comparison. Yellow peaks for S0, red peaks for S15. **(C)** Representative gel images for RT-qPCR results showing the expression of *MALAT1, NEAT1, XIST*, and *GAPDH* in S0 and S15. SM stands for size marker. **(D)** Representative gel images for RT-qPCR showing the ncRNA expression of *MALAT1, NEAT1, XIST, EGR1*, and *GAPDH* in S0 and S15 in the presence and absence of flavo. *EGR1*, a representative immediate early protein-coding gene, was included as a positive control whose expression is known to be induced by serum and to be regulated by P-TEFb. P-TEFb inhibition by flavo suppresses the expression of *MALAT1, NEAT1, XIST*, and *EGR1* upon serum induction. **(E)** RT-qPCR data showing RNA expression (relative expression values to DMSO S0) of *MALAT1, NEAT1, XIST, EGR1*, and *GAPDH*. DMSO-treated cells with serum induction for 0, 15, 30 min were labeled as D0, 15, 30; flavo-treated cells with serum induction for 0, 15, 30 min as F0, 15, 30. In DMSO controls, the expression of *MALAT1, NEAT1, XIST*, and *EGR1* was increased in response to serum induction (D15 and D30). In contrast, the expression of these genes was not induced by serum in the presence of flavo (F15 and F30). Note that the basal level (S0, shown as F0) of the ncRNAs, *MALAT1, NEAT1*, and *XIST* became increased in flavo-treated cells, even higher than F15 and 30. *EGR1* was included as a positive control and *GAPDH* as a reference gene and a negative control. Error bars, SEM (*n* = 3 biological replicates).

We noticed that *MALAT1, NEAT1*, and *XIST*, among the ncRNA genes, accumulate phospho-S2 Pol II upon serum stimulation (Figure 4A). *MALAT1* and *NEAT1* have been reportedly enriched in the active transcription loci (West et al., 2014). Another study has shown that *MALAT1* controls the G_1_/S cell cycle transition (Tripathi et al., 2013). In addition, *NEAT1* knockdown prevents cell proliferation to arrest laryngeal squamous cells in the G_1_ phase (Wang P. et al., 2016). Recently, increased *XIST* expression in osteosarcoma cells has suggested a new role of this ncRNA in cell proliferation (Yang et al., 2018). Our data are consistent with these reports and yet add additional information that these ncRNAs are induced in the early G_1_ phase. We termed these ncRNAs, which are expressed in the early G_1_ phase and regulate the cell-cycle progression, as immediate early ncRNAs. Genomic views of these genes in Figure 4B showed the increased occupancy of phospho-S2 Pol II upon serum induction (S0 versus S15). *XIST* is located embedded in TSIX. We note that phosphorylated S2 Pol II was enriched specifically in *XIST* (Supplementary Figure 1) but not in *TSIX*, suggesting its competitive expression as known for X-chromosome regulation (Gayen et al., 2015). Flavo treatment demolished the accumulation of S2 phospho-Pol II in *MALAT1, NEAT1*, and *XIST*, in contrast to the negative control, *DDX11-AS1* (Figure 4B). This inhibitory effect by flavo was comparable with established immediate early protein-coding genes, *EGR1, JUN*, and *FOS* that are regulated by P-TEFb for gene activation (Figure 4B).

Next, the transcriptional activation and expression of *MALAT1, NEAT1*, and *XIST* in the early G_1_ phase were examined using the reverse transcription quantitative PCR (RT-qPCR) analysis. Total RNAs were extracted from HEK293 cells after 18.5 h-serum starvation (arresting at G_0_ phase, S0) followed by 15- or 30-min serum induction (progressing to the G_1_ phase, S15). cDNA was constructed and then each ncRNA was quantified using a pair of primers targeting the mature ncRNA transcript. Consistent with the phospho-S2 Pol II ChIP-seq, the RT-qPCR results indicate that the transcription of *MALAT1, NEAT1*, and *XIST* is activated, and thus these genes become more actively expressed in S15 and S30, compared with S0 (Figure 4C–E). In addition, the expression of these genes was compared in the presence and absence of flavo using RT-qPCR analyses. As described above, HEK293 cells were treated with flavo at 1 μM final concentration for 1 h before serum induction and during the 15- or 30-min serum induction. The RT-qPCR results showed that flavo treatment interfered with the induction of these ncRNAs in S15 and S30, in contrast to DMSO controls (Figure 4C–E). Interestingly, it is noted that the basal level of *MALAT1, NEAT1*, and *XIST* was deregulated and dramatically increased in flavo-treated cells. This phenomenon is observed with the three ncRNAs, yet not with *EGR1*, a control protein-coding gene that is induced by serum and is positively regulated by P-TEFb. These results suggest that the kinase activity of P-TEFb is required for controlled gene induction and transcriptional activation of *MALAT1, NEAT1*, and *XIST*.

**FIGURE 5:**
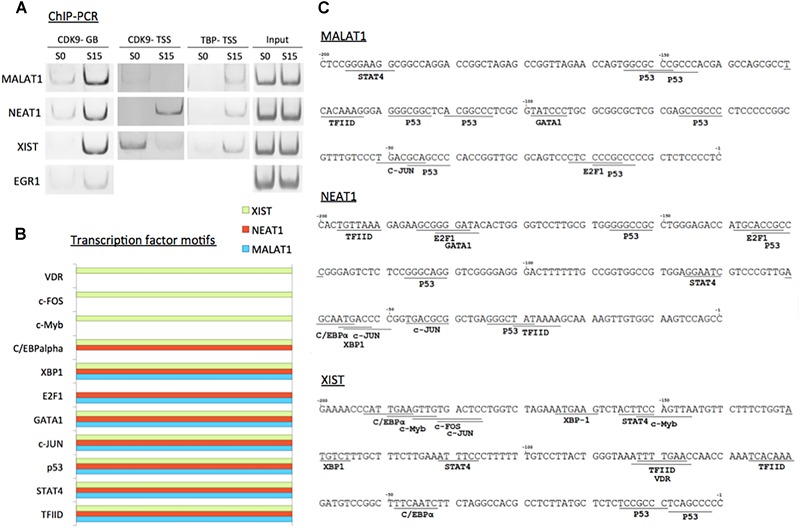
P-TEFb regulates transcriptional activation of ncRNA genes. **(A)** ChIP-PCR data showing the recruitment of CDK9 and TBP on *MALAT1, NEAT1*, and *XIST* upon serum induction in HEK293 cells. *EGR1* was used as a positive control. GB stands for gene body. While CDK9 recruitment in TSSs is variable among these three ncRNA genes, CDK9 is invariably increased in the gene bodies upon transcriptional activation using serum induction. **(B)** Transcription factor-binding motifs found in the promoters between –300 and –1 from the TSSs of *MALAT1* (light blue), *NEAT1* (red), and *XIST* (green). **(C)** Transcription factor-binding motifs (>85% consensus to the established canonical sequence) in the promoter (–300 to –1 from the TSS) of the three immediate early ncRNA genes.

We validated the function of P-TEFb to enhance the expression of *MALAT1, NEAT1*, and *XIST* using ChIP-PCR analysis. CDK9 was monitored in serum-starved (S0) and -stimulated HEK293 cells (S15). The results showed that CDK9 is recruited to the gene body of the three genes upon serum-induced transcriptional activation similar to the positive control, *EGR1*, a known immediate early protein-coding gene (Figure 5A). In addition, TBP, one of the subunits of TFIID, is a general transcription factor that is important for almost all mRNA and some tRNA transcriptions and stabilizes the pre-initiation complex (Huisinga and Pugh, 2007). However, TBP function in ncRNA genes has not yet been established. Therefore, we questioned whether TBP is involved in the expression of these genes. In Figure 5A, our ChIP-PCR analysis showed that TBP is recruited to the activated promoters of *MALAT1, NEAT1*, and *XIST*.

To learn potential transcriptional regulators for these ncRNA genes, we utilized a promoter motif search engine^2^. For each gene, the promoter region, -300 to -1 from TSS, was included to identify transcription factor binding motifs on the DNA primary sequence (Supplementary Figure 2). The resultant transcription factor-binding motifs with over 85% homologies with consensus sequences are listed in Figure 5B. The motifs that are commonly observed in all these lncRNA genes include TFIID as expected from our analysis with TBP (Figure 5A–C). Other motifs commonly found in *MALAT1, NEAT1*, and *XIST* are STAT4, p53, c-JUN, GATA1, and XBP1-binding motifs (Figure 5C). In addition, the E2F1-binding motif was found in *MALAT1* and *NEAT1*, and a transcriptional silencer, C/EBPα in *NEAT1* and *XIST*. c-Myb, c-FOS, and VDR were found in *XIST* (Figure 5B,C).

To summarize, our data suggest that the transcription of a large number of ncRNA genes is regulated by P-TEFb for S2 Pol II phosphorylation. CDK9 and HEXIM1 of the P-TEFb complex are engaged with a number of ncRNA genes. P-TEFb inhibition by flavo reduces phospho-S2 Pol II in the gene body of the majority of ncRNA genes, interfering with the progressive Pol II elongation. In addition, P-TEFb inhibition results in an overall increase of phospho-S2 Pol II density in the TSS of ncRNA genes. *MALAT1, NEAT1*, and *XIST* are the representative genes that are expressed in the immediate early cell-cycle progression from the G_0_ to G_1_ phase, and they require the function of CDK9 of P-TEFb for the regulation of gene induction and transcriptional activation and recruit TBP for active transcription.

## Discussion

In this study, we attempted to elucidate the mechanism of transcriptional regulation at ncRNA genes in human cells because the general structure of ncRNA transcription needs to be further established. In particular, we focused on the fact that many lncRNA genes harbor Pol II pausing and are stimulus-inducible (Bunch et al., 2016; Bunch, 2018). This led us to investigate the pausing and pause release mechanism in ncRNA genes. Since the discovery of Pol II promoter-proximal pausing in protein-coding genes (Rougvie and Lis, 1988; Core et al., 2008; Nechaev et al., 2010; Rahl et al., 2010; Bunch, 2017), quite a few protein factors, such as P-TEFb, NELF, DSIF, MYC, GAF, and PARP, have been identified to regulate it (Lee et al., 2008; Petesch and Lis, 2008; Rahl et al., 2010; Adelman and Lis, 2012; Lu et al., 2016). We have also recently identified a new Pol II pausing regulatory mechanism where DNA break and damage response signaling—including the factors, TRIM28, ataxia-telangiectasia mutated (ATM), DNA-dependent protein kinase (DNA-PK), and γH2AX and DNA topology factor, topoisomerase II—are activated and important for the pause release (Bunch et al., 2014, 2015; Bunch and Calderwood, 2015; Bunch, 2016; Bunch, 2017). Among these proteins, P-TEFb is the key factor to release Pol II from the pause, a hallmark for processive elongation (Adelman and Lis, 2012; Chen et al., 2018). P-TEFb essentially phosphorylates Pol II and negative regulators such as NELF and DSIF (Ping and Rana, 2001; Lu et al., 2016). These phosphorylation events appear to be the determinants for the paused gene transcription and expression. Therefore, we investigated the function and significance of P-TEFb in Pol II elongation and the gene activation in ncRNA genes.

Our major finding is that transcription in a number of inducible ncRNA genes employs the phosphorylation of Pol II CTD at S2 by P-TEFb (Figure 1). This is reminiscent of paused protein-coding gene activation and is consistent with the recent finding that enhancer RNA transcription resembles the transcription of protein-coding genes (Henriques et al., 2018). The apparent discrepancy is, however, that P-TEFb inhibition by flavo shows an interesting biphasic effect in ncRNA genes. Phosphorylation of S2 is dramatically increased in the TSSs, whereas it is overall decreased in the gene bodies (Figure 3). From the references and our data with protein-coding gene studies, we originally hypothesized that the P-TEFb inhibitor, flavo, would reduce the population of S2 Pol II in the TSSs (as well as in gene bodies). This was because Pol II CTD phosphorylation at serine 2 by P-TEFb develops in the early transcriptional elongation step immediately after transcriptional activation in protein-coding genes (Lis et al., 2000; Bunch et al., 2015; Aj et al., 2016). How S2 Pol II occupancy could be increased with diminished P-TEFb function before and during transcriptional activation at many ncRNA genes is unclear. We conjecture that S2 Pol II accumulates in the TSSs because it might be unable to translocate without P-TEFb function. Then, the increased S2 Pol II counts without functional P-TEFb may imply an additional kinase to phosphorylate serine 2 of Pol II CTD in the TSSs of ncRNA genes or may be attributable to the accumulation of pre-existing S2 Pol II that is unable to proceed to the gene body. Regarding these points, a few studies have reported that P-TEFb is mainly a CTD-serine 5 kinase (Zhou et al., 2000; Czudnochowski et al., 2012; Itzen et al., 2014). Because serine 5-phosphorylation is a pre-requisite of Pol II elongation, blocking it can simultaneously reduce S2 Pol II in the gene body as a consequence, as shown in the presence of flavo. In the future, addressing the nature of the overall increase in S2 Pol II in the TSSs of ncRNA genes in the presence of flavo seems crucial.

Our data show that the kinase and inhibitory subunits, CDK9 and HEXIM1, respectively are enriched in ncRNA genes, displaying large peaks in the TSSs. Importantly, these peaks are overlapped with Pol II peaks in ncRNA genes (Figure 2). This suggests that a number of ncRNA genes are engaged with Pol II and P-TEFb in the promoter-proximal site, consistent with the previous finding that ncRNA genes are regulated by Pol II pause and pause release (Bunch et al., 2016; Bunch, 2018). Since P-TEFb is recruited or de-repressed by transcriptional activators in the promoter of protein-coding genes, the expression of a large number of ncRNA genes could be presumably inducible. This also stresses the importance of promoter and promoter-proximal elements of ncRNA genes. For example, P-TEFb is recruited to *HSPA1B* by HSF1, a major transcriptional activator bound to the promoter of this gene upon heat shock (Lis et al., 2000; Zobeck et al., 2010). The example of P-TEFb de-repression for gene activation is shown in the transcription of HIV-1 genes. The HIV-1 TAR/TAT complex overcomes the inhibitory effect of HEXIM1 (Muniz et al., 2010; Aj et al., 2016). Factors such as HSF1 and TAT are nucleic acid-binding signal transducers that function in the upstream of P-TEFb. Seeing the abundant association of P-TEFb with the TSSs, it appears important to identify and understand the signal transduction molecules that provoke P-TEFb activation in the individual ncRNA gene. The enhancer components such as Mediator and eRNA reportedly interact with P-TEFb (Wang et al., 2013; Hertweck et al., 2016; Zhao et al., 2016). Therefore, it would be important to understand whether these enhancer elements collaborate with P-TEFb for ncRNA transcription in the future.

Lastly, three physiologically and clinically important lncRNAs, *MALAT1, NEAT1*, and *XIST*, were characterized. We have found that the expression of these lncRNA genes is activated in the early G_1_ phase and is dependent on P-TEFb (Figure 4). This is consistent with a few recent reports about these lncRNAs and how they tend to be upregulated in certain cancers and to control cell proliferation (Tripathi et al., 2013; Yildirim et al., 2013; Ma et al., 2015; Wang P. et al., 2016; Wang S. H. et al., 2016; Yang et al., 2018). It has also been reported that *MALAT1* and *NEAT1* regulate G_1_-S or G_2_-M transition and are found in actively expressing genes (Tripathi et al., 2013; West et al., 2014; Liu et al., 2017; Zhang et al., 2017). Our data suggest that *MALAT1* and *NEAT1* are expressed in the early G_1_ phase to modulate a variety of cell-cycle regulating genes, playing a critical role in cell growth. Without functional P-TEFb, the expression of these genes is not induced during the early G_1_ phase. Interestingly, however, P-TEFb inhibition noticeably increases the basal expression of these ncRNA genes (Figure 4). Although it is difficult to explain this phenomenon with current knowledge and without further investigation, we conjecture that it may attributable to some stress response by these ncRNA genes. We also ponder that it may indicate an uncharacterized role of P-TEFb during the resting state of ncRNA transcription. The representative immediate early genes including *EGR1, MYC, FOS*, and *JUN*, whose expression is dependent on P-TEFb, do not display the same phenomenon, and this might suggest a possible function of P-TEFb to suppress/moderate the expression of certain ncRNA genes during the transcriptional resting state. If this is the case, inhibition of P-TEFb would increase the basal expression level of these ncRNA genes as shown here. Validating these hypotheses to understand the phenomenon requires further investigation.

In addition, we found that TBP is recruited to the promoters of these lncRNA genes upon transcriptional activation. TBP binding to the promoter regulates the transcription initiation and noise (Ravarani et al., 2016). Mot1p competes with SAGA for TBP and suppresses TBP for antisense ncRNA transcription (Koster and Timmers, 2015; Ravarani et al., 2016). This suggests that ncRNA transcription is initiated by TBP binding as in protein-coding gene transcription. In addition, we note the couples of transcription factor-binding motifs including Myb, E2F1, and STAT4 in the promoters of *MALAT1, NEAT1*, and *XIST* (Figure 5). We anticipate further clarification of the promoter elements of the three lncRNA genes using molecular biology and biochemical analyses in the future. We propose that the transcriptional mechanisms of how these lncRNA genes are activated and how they further activate other genes are important to be understood on the molecular level.

To summarize, our study has shown that P-TEFb is associated with a number of ncRNA genes, and the activation of these genes is regulated by S2 Pol II phosphorylation by P-TEFb in humans. Intriguingly, P-TEFb inhibition noticeably increases S2 Pol II in the TSSs whereas it decreases S2 Pol II in the gene bodies upon transcriptional activation of ncRNA genes. Our previous and current data suggest that ncRNA and mRNA transcription are regulated mostly by similar mechanisms, while P-TEFb inhibition unexpectedly increases S2 Pol II phosphorylation in the TSSs of many ncRNA genes. We have identified *MALAT1, NEAT1*, and *XIST* as immediate early ncRNA genes and have validated that P-TEFb and TBP are recruited upon transcriptional activation. For the first time, to the best of our knowledge, P-TEFb was studied genome-wide and at the cellular molecular level for human ncRNA genes. It is believed that the functional engagement of P-TEFb in ncRNA transcription provides valuable directions for the understanding of the transcription system that governs the expression of a large number of ncRNA genes in metazoan cells.

## Author Contributions

JK, KK, SL, and HB performed bioinformatics and DNA sequence analyses. HB, HC, and SJ carried out RT-PCR experiments. DJ and D-HC performed flow cytometry and fluorescence-activated cell sorting assays. HB performed ChIP. HB designed the experiments and wrote the manuscript.

## Conflict of Interest Statement

The authors declare that the research was conducted in the absence of any commercial or financial relationships that could be construed as a potential conflict of interest.

## Footnotes

1 www.ensemble.org

2 http://alggen.lsi.upc.es/cgi-bin/promo_v3/promo/promoinit.cgi?dirDB=TF_8.3
