# Supplementary material for: P-TEFb Regulates Transcriptional Activation in Non-coding RNA Genes
Source: Front Genet. 2019 Apr 24;10:342. doi: 10.3389/fgene.2019.00342 (PMC6491683; doi:10.3389/fgene.2019.00342)
Supplement: Supplementary file 1 [file Data_Sheet_1.PDF]

SUPPLEMENTARY FIGURES

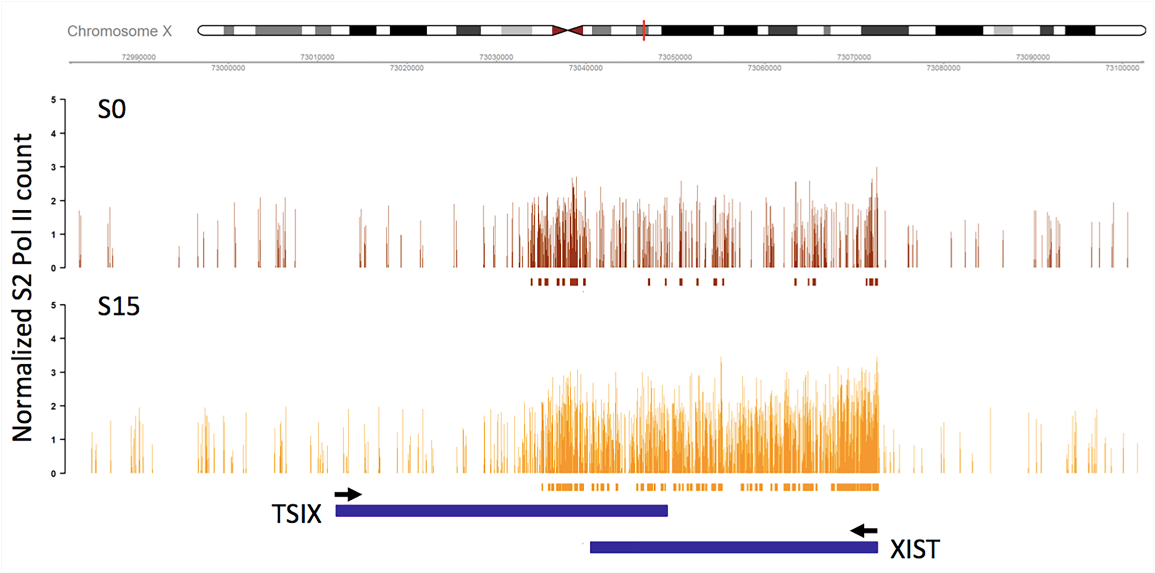

Supplementary Figure 1. Chromosomal view of *XIST* and *TSIX*. Normalized S2 Pol II counts from the ChIP-seq analysis are shown in the X chromosome locus of *XIST* and *TSIX*. The S2 Pol II peaks are increased at *XIST* but not in *TSIX* in the serum-induced HEK293 cells (S15), compared with the cells before serum induction (S0). TSS is marked with a black arrow.

a.

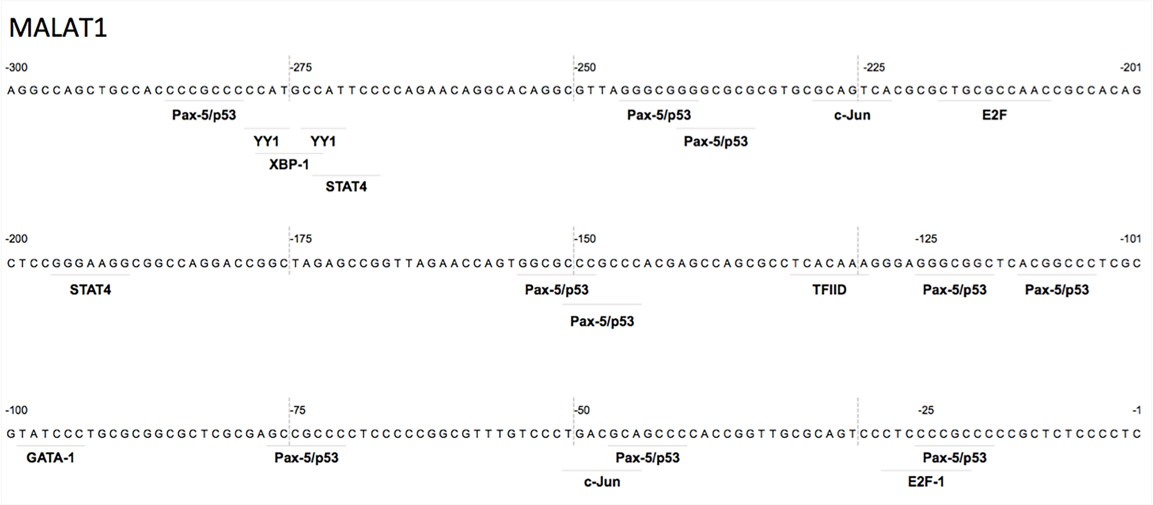
MALAT1
-300 -275 -250 -225 -201
AGGCCAGCTGCCACCCCGCCCCCATGCCATTCCCCAGAACAGGCACAGGCGTTAGGGCGGGGCGCGCGTGCGCAGTCACGCGCTGCGCCAACCGCCACAG
Pax-5/p53 Pax-5/p53 c-Jun E2F
YY1 YY1 Pax-5/p53
XBP-1
STAT4
-200 -175 -150 -125 -101
CTCCGGGAAGGCGGCCAGGACCGGCTAGAGCCGGTTAGAACCAGTGGCGCCCGCCCACGAGCCAGCGCCTCACAAAGGGAGGGCGGCTCACGGCCCTCGC
STAT4 Pax-5/p53 TFIID Pax-5/p53 Pax-5/p53
Pax-5/p53
-100 -75 -50 -25 -1
GTATCCCTGCGCGGCGCTCGCGAGCCGCCCCTCCCCCGGCGTTTGTCCCTGACGCAGCCCCACCGGTTGCGCAGTCCCTCCCCGCCCCCGCTCTCCCCTC
GATA-1 Pax-5/p53 Pax-5/p53 Pax-5/p53
c-Jun E2F-1

b.

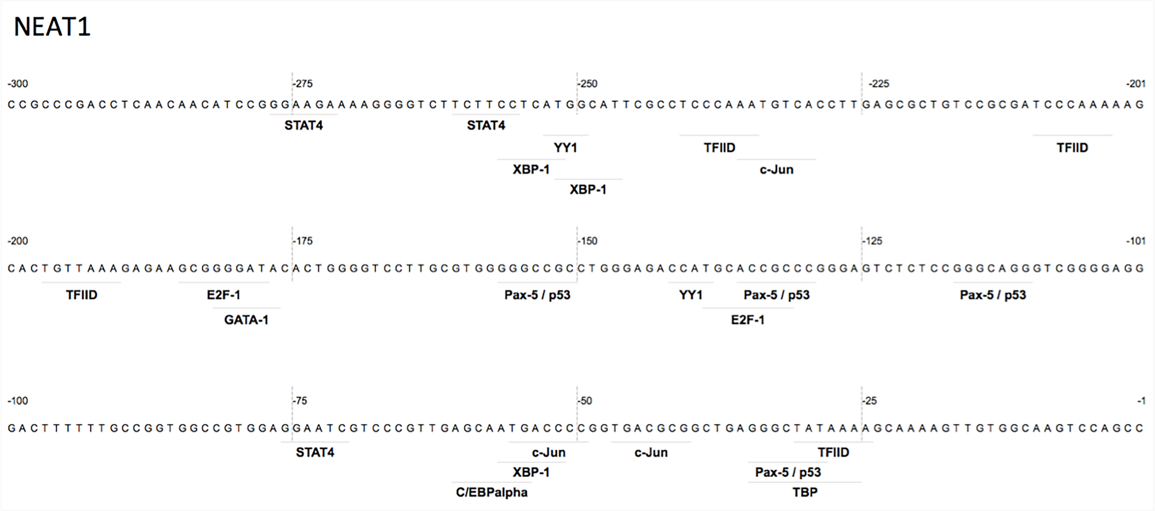
NEAT1
-300 -275 -250 -225 -201
CCGCCCGACCTCAACAACATCCGGGAAGAAAAGGGGTCTTCTTCCTCATGGCATTCGCCTCCCAAATGTCACCTTGAGCGCTGTCCGCGATCCCAAAAAG
STAT4 STAT4
YY1 TFIID TFIID
XBP-1 c-Jun
XBP-1
-200 -175 -150 -125 -101
CACTGTTAAAGAGAAGCGGGGATACACTGGGGTCCTTGCGTGGGGGCCGCCTGGGAGACCATGCACCGCCCGGGAGTCTCTCCGGGCAGGTCGGGGAGG
TFIID E2F-1 Pax-5 / p53 YY1 Pax-5 / p53 Pax-5 / p53
GATA-1 E2F-1
-100 -75 -50 -25 -1
GACTTTTTTGCCGGTGGCCGTGGAGGAATCGTCCCGTTGAGCAATGACCCCGGTGACGCGGCTGAGGGCTATAAAAGCAAAAGTTGTGGCAAGTCCAGCC
STAT4 c-Jun c-Jun TFIID
XBP-1 Pax-5 / p53
C/EBPalpha TBP

c.

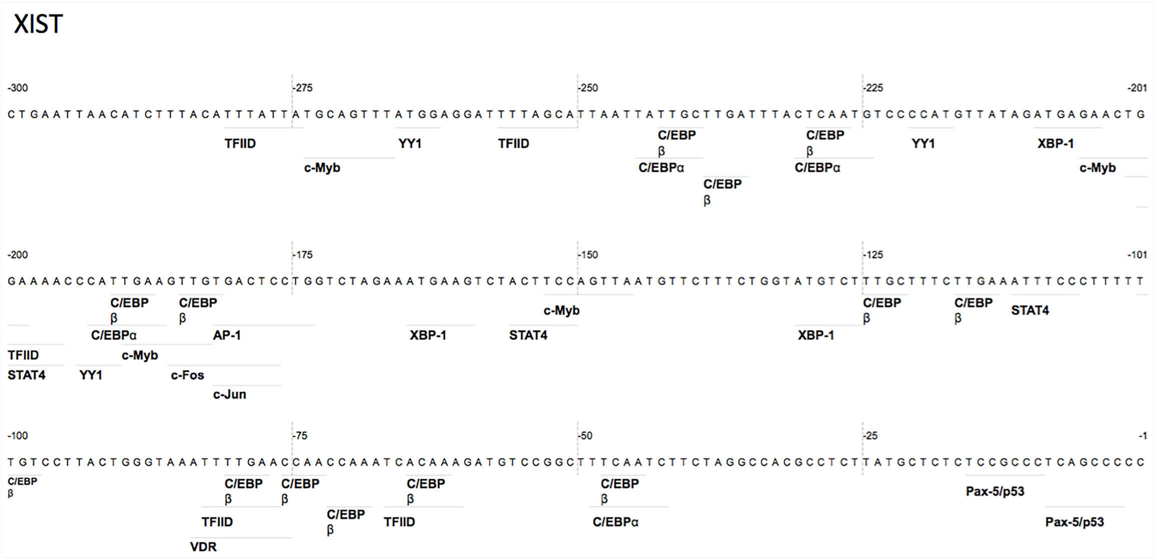

Supplementary Figure 2. Transcription factor-binding motifs at *MALAT1*, *NEAT1*, and *XIST*. Transcription factor-binding motifs with > 85% consensus are shown between –300 to –1 from the TSS of each ncRNA gene. (**a**) *MALAT1*. (**b**) *NEAT1*. (**c**) *XIST*.

Supplementary Table 1.

The sequences of oligos used in this study

**For ChIP-PCR Assay**	
**MALAT1 Promoter Forward**	CCT GAC GCA GCC CCA CCG
**MALAT1 Promoter Reverse**	CGA GCT GAG GCT TCC CGG CGC C
**NEAT1 Promoter Forward**	GGG ATA CAC TGG GGT CCT TGC G
**NEAT1 Promoter Reverse**	GCT GGA CTT GCC ACA ACT TTT GC
**XIST Promoter Forward**	CCA ACC AAA TCA CAA AGA TGT CCG GC
**XIST Promoter Reverse**	GAG AGA TCT TCA GTC AGG AAG CTT CCA GCC C
**EGR1 Gene Body Forward**	GCT TTC CCG GCC CAG GTC AGC AGC TTC CCT TCC
**EGR1 Gene Body Reverse**	CTT TTT CTC CCT TTT CCC TTT CTT TCC CCT TTC CC
**MALAT1 Gene Body Forward**	GGG AAA GGG GGA AAG CGG GCA ACC
**MALAT1 Gene Body Reverse**	CCA GTC TAC AAG TTA CAT GTT CCC ACC C
**NEAT1 Gene Body Forward**	CCC TGG CCT GAC ATG TGT GTC CCT GAG C
**NEAT1 Gene Body Reverse**	CCC CAG GAC CCA AAG GTA CAC AAA GC
**XIST Gene Body Forward**	GGG CAC CTT CTC ATG GAC TCC CTT TGC
**XIST Gene Body Reverse**	GGC ATC CTT GTC TAG GGC ACA AGA ACC
**For RT-PCR analysis**	
**MALAT1 Forward**	GGG AAA GGG GGA AAG CGG GCA ACC
**MALAT1 Reverse**	CCA GTC TAC AAG TTA CAT GTT CCC ACC C
**NEAT1 Forward**	CCC TGG CCT GAC ATG TGT GTC CCT GAG C
**NEAT1 Reverse**	CCC CAG GAC CCA AAG GTA CAC AAA GC
**EGR1 Forward**	GCT TTC CCG GCC CAG GTC AGC AGC TTC CCT TCC
**EGR1 Reverse**	CTT TTT CTC CCT TTT CCC TTT CTT TCC CCT TTC CC
**GAPDH Forward**	AGA AGG CTG GGG CTC ATT TG
**GAPDH Reverse**	AGG GGC CAT CCA CAG TCT TC
**XIST Forward**	GGG CAC CTT CTC ATG GAC TCC CTT TGC
**XIST Reverse**	GGC ATC CTT GTC TAG GGC ACA AGA ACC
